# Sensor for sports applications: monitoring and assessment of intrinsic capacity enhancement in older adults during brisk walking

**DOI:** 10.3389/fpubh.2025.1659600

**Published:** 2025-09-11

**Authors:** Fangyuan Ju, Xu Han, Mengyun Zhao, Shuo Wang

**Affiliations:** ^1^Department of Physical Education, Yangzhou University, Yangzhou, China; ^2^Department of Physical Education, Gansu University of Political Science and Law, Lanzhou, China

**Keywords:** sensors, microchips, wisdom sports, wearable technology, older adults fitness

## Abstract

**Background:**

As population aging accelerates, the development of precise health monitoring technologies for older adults is crucial for mitigating functional decline and chronic disease risks. The “Intrinsic Capacity (IC)” framework, proposed by the World Health Organization(WHO), defines five core dimensions of older adults' functional ability: locomotion, vitality, cognition, psychological and sensory. Wearable motion sensors provide a novel approach for early detection and continuous monitoring of these dimensions.

**Methods:**

This study conducts a systematic literature review of empirical research in 20 years (from 2005 to 2025), focusing on how motion sensors capture IC-related changes during brisk walking in older adults. A total of 23 studies were included after screening.

**Results:**

Key findings reveal that adults aged 60–74 demonstrate the highest levels of technology acceptance and compliance, whereas individuals over 80 years old favor simpler, more user-friendly devices. Triaxial accelerometers, pressure sensors, photoplethysmography (PPG), and electrodermal activity (EDA) sensors are used to monitor gait rhythm, stability, heart rate regulation, and emotional stress, respectively.

**Conclusions:**

The results indicate that motion sensor technologies offer comprehensive coverage across all five IC dimensions and hold strong potential for continuous assessment, anomaly detection, and personalized intervention. Future research should prioritize multimodal sensor integration and algorithm optimization to enhance real-world applications in health management and remote monitoring for aging populations.

## 1 Background

With global population aging, maintaining physiological function and independent living in older adults has become a major focus of public health and clinical research (1, 2). The World Health Organization (WHO) introduced the concept of “intrinsic capacity (IC),” which refers to an individual's composite physical, cognitive, and psychological functioning (3–5). This framework has been widely adopted to assess health potential and functional reserve in older adults (6, 7). IC consists of five core dimensions: locomotion, vitality, sensory function, cognition, and psychological state (5, 8). Unlike traditional assessments based on disease diagnosis or functional scales, the IC framework emphasizes continuous functional change and highlights the urgent need for high-resolution, non-invasive, multidimensional assessment tools (5, 6, 9).

Among various lifestyle behaviors, brisk walking is regarded as an ideal activity for reflecting IC status because it engages neural, muscular, sensory, and cognitive systems simultaneously (10, 11). Empirical studies have shown that brisk walking improves lower limb strength, balance, and cardiorespiratory endurance, and helps regulate blood pressure and glucose levels, thereby reducing fall risk (12, 13). Walking also involves attentional control, rhythmic regulation, and perceptual feedback, with behavioral characteristics closely linked to multiple IC dimensions (11, 14). For example, gait rhythm fluctuations may indicate cognitive load, delayed movement initiation may reflect reduced vitality, and rapid adaptation to terrain may signal efficient sensory processing (15, 16). Walking is thus not only an effective intervention but also a valuable window into multidimensional IC assessment.

Motion sensor technology provides a key means of objectively quantifying IC's multidimensional functions during walking (17, 18). In recent years, wearable and ambient sensors—such as inertial measurement units (IMUs), plantar pressure sensors, hear trate variability (HRV)sensors, EDA, modules and PPG modules—have been widely adopted to monitor older adults‘ health behaviors (19, 20). These devices capture gait parameters, movement patterns, reaction times, and physiological rhythms with high frequency and low noise, mapping them to IC dimensions through feature extraction and modeling (16, 21, 22). Technological interventions have been shown to enhance older adults' sense of control and achievement, while supporting cognitive self-monitoring, emotional regulation, and sustained autonomy (11, 23). Therefore, developing motion sensor–based IC assessment systems represents a promising direction for digital health and precision aging interventions.

This review aims to systematically examine how motion sensors support walking in older adults, focusing on population-specific adaptations, sensor mechanisms, and technical capabilities for monitoring the five dimensions of intrinsic capacity.

## 2 Research method

### 2.1 Search strategy

This study adopted a systematic literature review approach and conducted a comprehensive search in four major databases: PubMed, Web of Science, EBSCOhost (SPORTDiscus & CINAHL), and IEEE Xplore, while Google Scholar was used as a complementary database for obtaining gray literature that may not be gray literature that may not be indexed in mainstream databases. The deadline for the search was 30 March 2025. Keywords searched included: motion sensors, “older Adults”, “brisk walking”, “ exercise monitor” and so on. During the search process, keyword combinations were used and Boolean logic operators (e.g., “AND”, “OR”, “NOT”) were applied to optimize the search terms, ensuring the comprehensiveness and accuracy of the search strategy. The specific search strategies are as follows.

#### 2.1.1 Keyword combination

Combine “Wearable Devices” and “Older Adults”, and use the Boolean operator “AND” to connect them, such as “Wearable Devices AND Older Adults”. At the same time, use the “OR” operator to connect related concepts, such as “Older adults OR Aging Population”.

#### 2.1.2 Exclusion criteria

The “NOT” operator was used to exclude literature that was not relevant to the topic, e.g., “NOT Children”, to ensure that the search results focused on the older adults population. For non-wearable interventions, “NOT Non-Wearable Interventions” was used to ensure consistency between the target population and the objectives of the study.

In addition, reference lists, citation searches, and manual searches were used to identify relevant literature that may have been missed. The initial search yielded 2,382 articles.

### 2.2 Inclusion and exclusion criteria

This study screened the literature based on the population, interventions, comparisons, findings and study design (PICOS) model. The specific criteria are shown in Table 1.

**Table 1:** PICOS framework.

**Acronym**	**Definition**	**Scopes**
P	Population	Older adults
I	Intervention	Use of wearable or environmental motion sensors (e.g., accelerometers, pressure sensors, PPG, EDA)
C	Comparison	No intervention, usual care, or interventions without sensor-based monitoring
O	Outcome	Changes in IC domains: locomotion, vitality, cognition, sensory, psychological status (measured via gait, HRV, EDA, activity level, etc.)
S	Study design	Systematic reviews of experimental studies

Exclusion criteria included 1. studies in non-older adults subjects (i.e., under 60 years of age) or interventions not related to walking; 2. studies that were not peer-reviewed or did not have the full text available; and 3. interventions involving only non-motor sensors or studies with no specific motor findings.

### 2.3 Literature screening process

**Figure 1:**
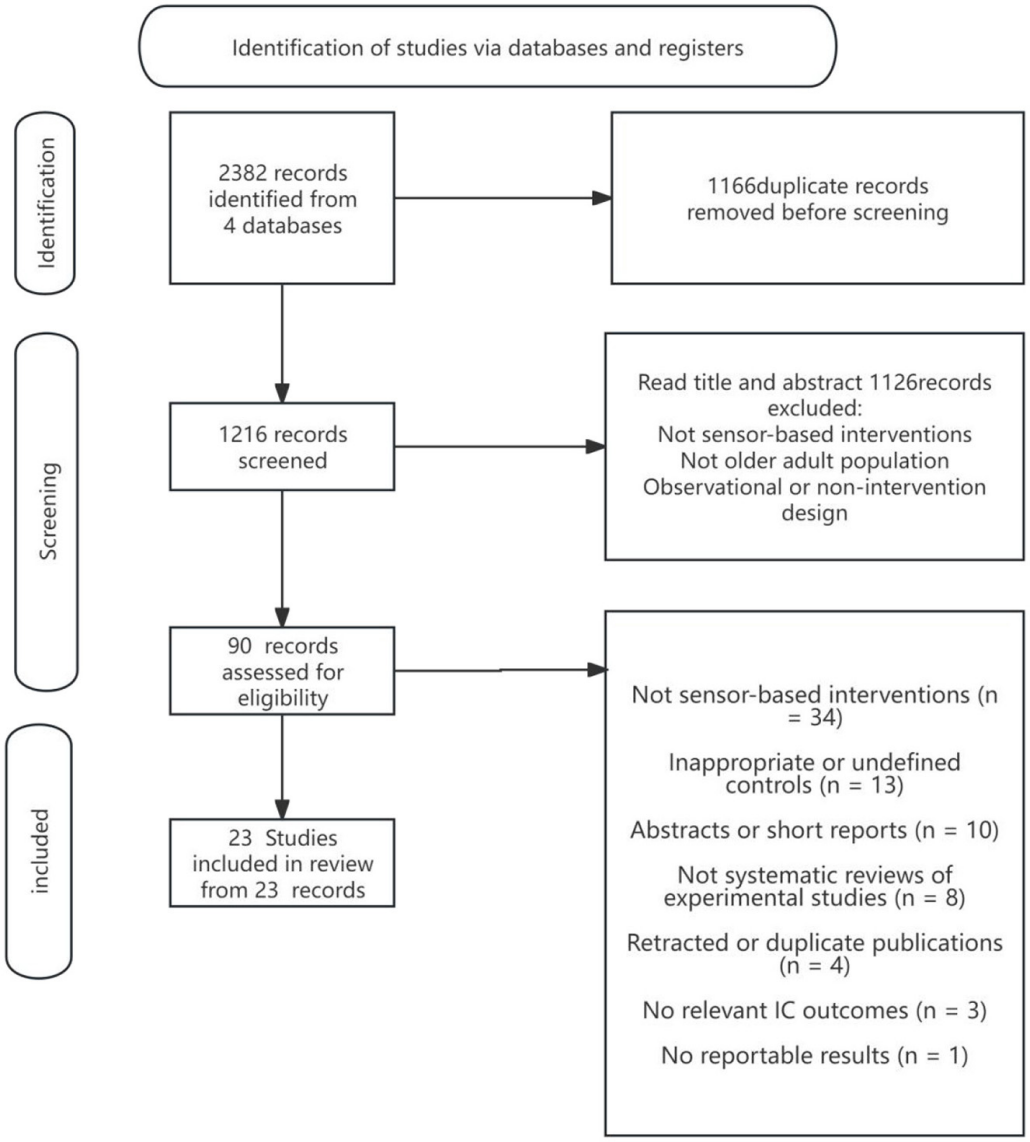
PRISMA flowchart.

The literature screening process in this study strictly followed the PRISMA 2020 standard and used the Rayyan Qatar Computing Research Institute (QCRI) literature management tool to ensure the objectivity and reproducibility of the screening. The screening process included the following steps: first, all relevant literature was extracted from various databases according to the search strategy; subsequently, EndNoteX9 was used to perform automatic de-duplication combined with manual screening to remove duplicates; at the title and abstract screening stage, two independent reviewers (Reviewer 1 & Reviewer 2) screened the literature according to the inclusion criteria; for literature that met the initial screening criteria, full-text reading screening was performed, and the literature was finally identified. In case of disagreement during the screening process, a third independent reviewer (Reviewer 3) made a decision. The complete screening process and screening results are detailed in Figure 1 (PRISMA flowchart) in the Results section.

### 2.4 Data extraction and analysis

Basic information about the study was extracted from the included literature, including the study population, the intervention, and the main results. Standard data forms were used to record the extracted information for summarization and analysis. This study mainly focuses on the evidence of the effectiveness of wearable sex devices in promoting exercise participation among older adults, and combines qualitative and quantitative analyses for a comprehensive discussion. Table 2 summarizes the relevant information from the 23 papers screened in this exercise.

**Table 2:** Characteristics of included studies (*n* = 23).

**Author**	**Population characteristics**	**Main sensors**	**Key findings**
Lu et al. (34)	Average age 60 with community-dwelling women	Accelerometer	Moderate-to-vigorous activity is associated with better physical function, while sedentary behavior correlates with decline
Nagayoshi et al. (25)	Older adults with health	Accelerometer	Estimated activity intensity from accelerometer agrees with indirect calorimetry, supporting cross-age applicability
Nakajima et al. (22)	Older adults with independent walking ability	Plantar pressure sensor	Gait parameters such as pressure path and step length effectively assess gait stability in older adults
Asher et al. (41)	Older adults with health	PPG, plantar pressure sensor, accelerometer	Synchronization of gait and heart rate reveals individual physiological-behavioral rhythms for risk modeling
Bollaert et al. (36)	Older adults with multiple sclerosis and healthy controls	Accelerometer	MS group showed lower activity, prolonged sitting, and functional decline, supporting need for tailored intervention
Delmastro et al. (23)	Older adults with mild cognitive impairment and frailty	EDA, HRV	Machine learning system identifies stress and training states, suitable for cognitive health monitoring inMild Cognitive Impairment(MCI)
Master et al. (46)	Average age 65 with knee osteoarthritis	Tri-axial accelerometer	Physical function test (e.g., 400 m walk) predicts achieving 6,000 steps/day, supporting clinical goal-setting
Song et al. (39)	Older adults with weak foot and fall risk	Plantar pressure sensor	Wearable pressure data based on weak foot characteristics effectively assess fall risk and support intervention
Lee et al. (30)	Average age 74.5 with health	Plantar pressure board sensor	GEMS improved step speed, stride length, and stance time; reduced EMG activity and improved plantar pressure distribution
Howcroft et al. (32)	Older adults with dual-task gait testing	Plantar pressure + accelerometer	Dual-task condition impacts gait stability; sensor data support fall risk assessment
Mori et al. (43)	Older adults with walking training assistance	PPG	Robot with integrated PPG accurately estimates exercise intensity with < 1 bpm error
Kakita et al. (37)	Older adults with cardiovascular disease	Accelerometer	Most activity is non-exercise based, emphasizing the value of daily physical activity assessment
Soares-Miranda et al. (42)	Older adults aged >65 from the Cardiovascular Health Study	24-h Holter electrocardiography(ECG)	Physical activity is positively associated with HRV, especially MVPA with HF HRV, indicating benefit to autonomic nervous health
Kocuvan et al. (33)	Older adults with simulated fall gait	Accelerometer	Wrist-worn device achieves 86% classification accuracy via Support Vector Machine(SVM), outperforming phone (73%) for fall risk prediction
Kumar et al. (38)	Average age 65 with prefrail and frail status	Accelerometer	60-s continuous walking data identify frailty status with 76.8% sensitivity and 80% specificity
Yu et al. (24)	Average age 78.9 with patients and healthy individuals	PPG	RGB camera estimates HR with RMSE of 0.48 bpm; HRV frequency correlation up to 0.7; suitable for non-invasive monitoring
Nath et al. (26)	Older adults with stress and cortisol monitoring	EDA, PPG	Multi-modal + cortisol-based XGBoost model achieves 87% accuracy for stress detection in older adults
Mickle et al. (28)	Average age 70 with community-dwelling	Plantar pressure sensor	Foot pain and high plantar pressure correlate with fall risk; intervention may help reduce falls
Lai et al. (109)	Average age 70 with independent walking ability	Accelerometer	Total MVPA duration more strongly linked to physical function than specific activity periods
Clarke et al. (21)	Older adults with functional impairment and walking aids	Accelerometer	AX3 effectively monitors walking activity, with high validity in functionally impaired older adults
Karthikeyan et al. (29)	Older adults with gender-balanced sample	HRV	Multi-modal neurophysiological indicators accurately detect cognitive stress (accuracy 78–98%), sensitive to gender/activity
Choi et al. (44)	Older adults living alone (*N* = 14), non-depressed	EDA, PPG, accelerometer	Machine learning model predicts depressive mood with recall of 82.7%, suitable for passive monitoring
Zhang et al. (31)	Older adults with and without fall history	Plantar pressure insole, accelerometer	Machine learning on multi-modal data achieves >85% fall prediction accuracy; suitable for daily use in older adults

## 3 Results

### 3.1 Target audience

Of the 23 studies included in this systematic review, all of them explicitly identified older adults as the core target audience for exercise bracelet interventions, although there were significant differences in how the concept of “older adults” was defined, the enrolment criteria, and the health status stratification. This reflects the fact that there are still inconsistencies in the theoretical basis and practical standards in the process of target user modeling in the current study.

#### 3.1.1 Differences in the use of different ages of older adults

Age-related differences in physiological characteristics and motor abilities among older adults have a direct impact on the signal acquisition quality and functional stability of motion sensor systems (24). In particular, among advanced-age populations, the deterioration of gait rhythm, circulatory system function, and skin electrodermal responsiveness often results in distorted sensor inputs and degraded output data quality. These changes place greater demands on the adaptability and robustness of system-level monitoring architectures (25–27).

In the younger-old age group (60–74 years), triaxial accelerometers, PPG sensors, and EDA modules generally perform within standard operational parameters (21, 24, 26, 28, 29). Gait amplitude and movement intensity continue to elicit distinct peak acceleration signals; the PPG sensor generates stable pulse waveforms during short resting periods; and the EDA channel exhibits effective responses to emotional stimuli and cognitive task loads (24, 26). This cohort has demonstrated high device compliance and favorable physiological responsiveness in existing studies, enabling low-latency algorithm execution and high recognition accuracy across sensor platforms.

In contrast, sensor systems deployed in the oldest-old group (≥85 years) or among frail individuals often encounter significant performance constraints from both the signal source and user interaction levels. For accelerometers, reduced stride amplitude and frequency lead to smoothed waveform outputs, particularly attenuating anterior–posterior (X-axis) and lateral (Y-axis) signal peaks, which in turn increase errors in gait cycle segmentation and threshold-triggered event detection (25). For PPG sensors, reduced skin permeability and age-related decline in microcirculation and arterial elasticity flatten the pulsation waveform, compromising the accuracy of key parameters such as heart rate recovery Heart Rate Recovery (HRR) and HRV (24). Similarly, EDA signals are weakened due to thickened stratum corneum and diminished sweating response, significantly impairing sensitivity to emotional stress detection (26).

In summary, the heterogeneity of physiological expression and physical capacity across different older adults age groups critically affects the signal acquisition efficiency of core sensors—including accelerometers, PPG, and EDA modules—and diminishes the reliability of output data. Older adults are more susceptible to signal attenuation, waveform instability, and decreased recognition accuracy, which in turn limits the effectiveness of standard algorithms and conventional hardware designs. To address these limitations, developing a stratified evaluation framework based on age-specific physiological characteristics has emerged as a key engineering direction for enhancing system robustness and improving the precision of sensor-based interventions.

#### 3.1.2 Functional requirement focus of older adults with different health status

A total of 8 out of 23 included studies explicitly qualified the health status of the subjects, reflecting the critical impact of the health level of older adults on the deployment strategy of sensor systems. Individuals in different functional states have systematic differences in perceptual metrics, device acceptance, and data accuracy requirements, which should be addressed with fine stratification.

Among them, five focused on older adults with debilitated function or high risk of falling, and commonly adopted a synergistic solution of lumbar IMUs and plantar pressure sensors for monitoring postural stability, gait symmetry, and abnormal gait acceleration (28, 30, 31). Studies have shown that the coefficient of variation (CV) of gait is significantly higher in this group in complex terrain or dual-task conditions, and sensor deployment requires high sampling frequency and sensitive anomaly recognition (21, 32). For older adults with chronic medical conditions (e.g., cardiovascular disease, diabetes, etc.), studies have typically used multimodal devices integrating PPG, HRV, and triaxial acceleration to provide comprehensive monitoring of locomotor center of motion dynamics, recovery rate, and physiological stress levels (21, 24, 33). These devices assist individuals with pacing and load self-awareness through physiological threshold feedback. In contrast, healthy older adults using lightweight, low-intervention accelerometer bracelets for daily behavioral tracking and interactive motivation, mainly monitoring moderate-vigorous intensity physical activity (MVPA) time and average daily step count. Feedback was based on APP notifications or vibration alerts, with emphasis on the establishment of behavioral habits and exercise continuity (25, 33, 34).

In summary, there are significant differences in the deployment of sensors by older adults in different health states. The frail individuals rely more on posture and stability monitoring, the chronically ill need physiological load sensing support, and the healthy group focuses on behavioral recording and interactive feedback. Sensor interventions should be based on health stratification to build a functional matching model to achieve optimal fit and intervention efficiency.

### 3.2 Differential effects of sensor types on walking exercise monitoring

#### 3.2.1 Triaxial accelerometers for step counting and intensity recognition

**Figure 2:**
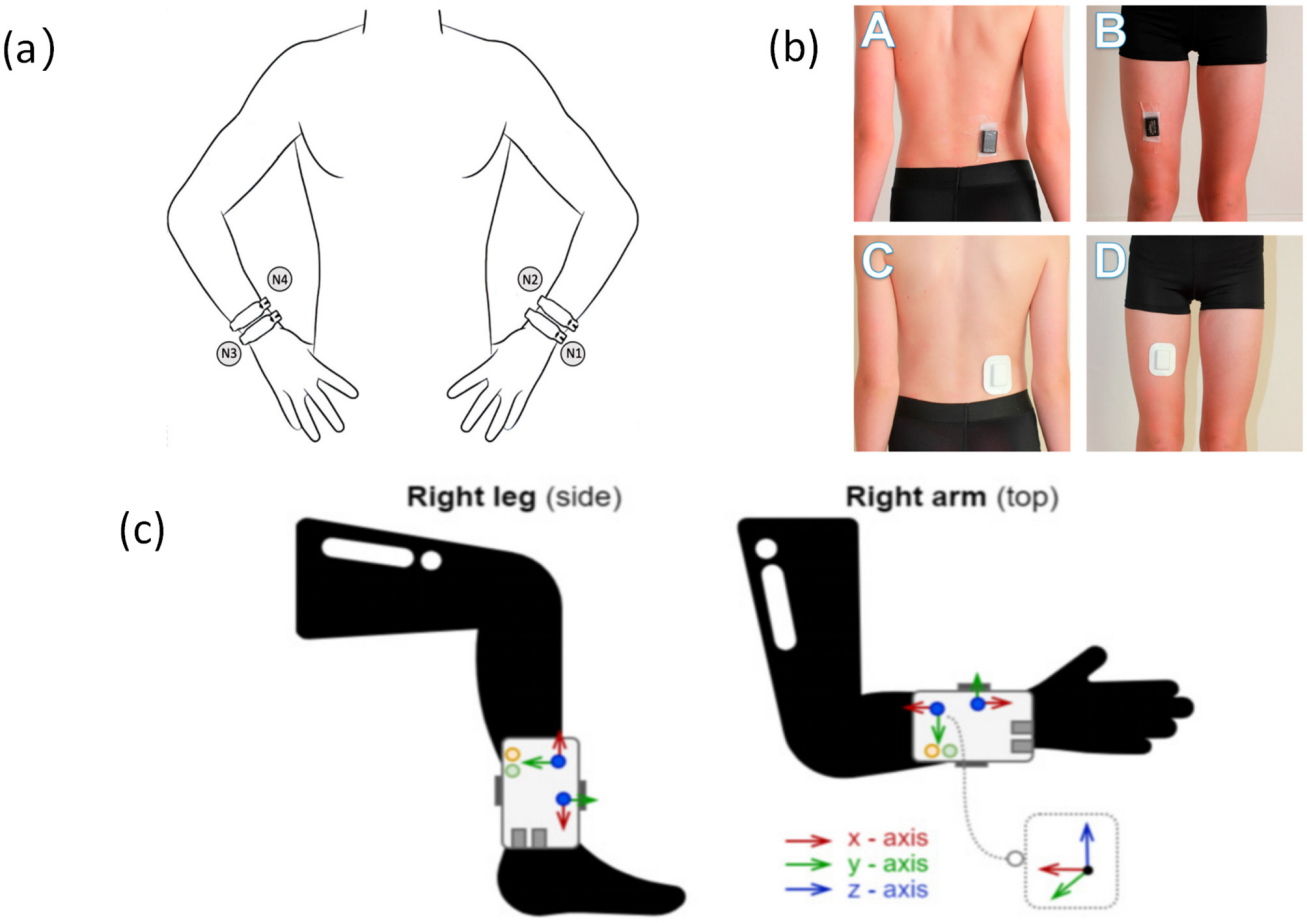
Accelerometers are placed at different locations on the body **(a)**. Accelerometer placement and wrist location (47), copyright (2025), with permission from MDPI. **(b)** Accelerometers are located at the waist and thighs (104), copyright (2018), with permission from MDPI. **(c)** Accelerometers are placed on the ankles and arms (105), copyright (2021), with permission from MDPI.

As a core module of IMUs, triaxial accelerometers utilize MEMS microstructures to detect linear acceleration along the X, Y, and Z axes. They are widely used in older adults walking monitoring systems due to their compact size, low power consumption, and clear signal structure, enabling non-invasive, continuous tracking of daily movement patterns (21, 25, 35) (refer to Figure 2). These sensors are particularly suitable for monitoring low-intensity activities in older adults.

Among the 23 studies reviewed, 10 utilized triaxial accelerometers as the primary monitoring sensor to derive multidimensional behavioral parameters such as step count, moderate-to-vigorous physical activity (MVPA) duration, total energy expenditure (EE), and daily activity patterns (25, 33, 34). During the intervention period, participants' average daily step count increased by 1,120–1,920 steps, MVPA duration rose by 16.2%−31.7%, accompanied by simultaneous improvements in self-efficacy and adherence (33, 35–37). These indicators are derived from acceleration data, classified using time-window segmentation and dynamic threshold algorithms, demonstrating good temporal sensitivity and quantitative reliability.

Mainstream devices typically operate at a sampling frequency of 30–100 Hz, with a dynamic range of ±6 g to ±8 g, and apply first-order low-pass filters to suppress high-frequency noise (25, 33, 35). At the algorithmic level, features such as mean, standard deviation, and signal magnitude area (SMA) are extracted within sliding windows, and motion recognition models are developed using dynamic thresholding or combined angular velocity–acceleration detection (31, 38). Some platforms further integrate posture estimation and position classification modules to enhance the detection of marginal behaviors such as standing, turning, and slow walking (33). By incorporating wear-time tracking and signal-loss compensation mechanisms, these systems can collect comprehensive daily behavioral trajectories, supporting remote management and follow-up interventions.

In summary, triaxial accelerometers serve as fundamental sensing units in wearable motion monitoring systems, supported by mature engineering configurations and data processing frameworks. Future integration of sensors such as EDA and PPG for multimodal data fusion, along with the adoption of deep learning-based micro-behavior recognition algorithms, is expected to enhance resolution in abnormal behavior detection, personalized exercise prescription, and dynamic load management for older adults.

#### 3.2.2 Plantar pressure sensors for gait stability assessment

**Figure 3:**
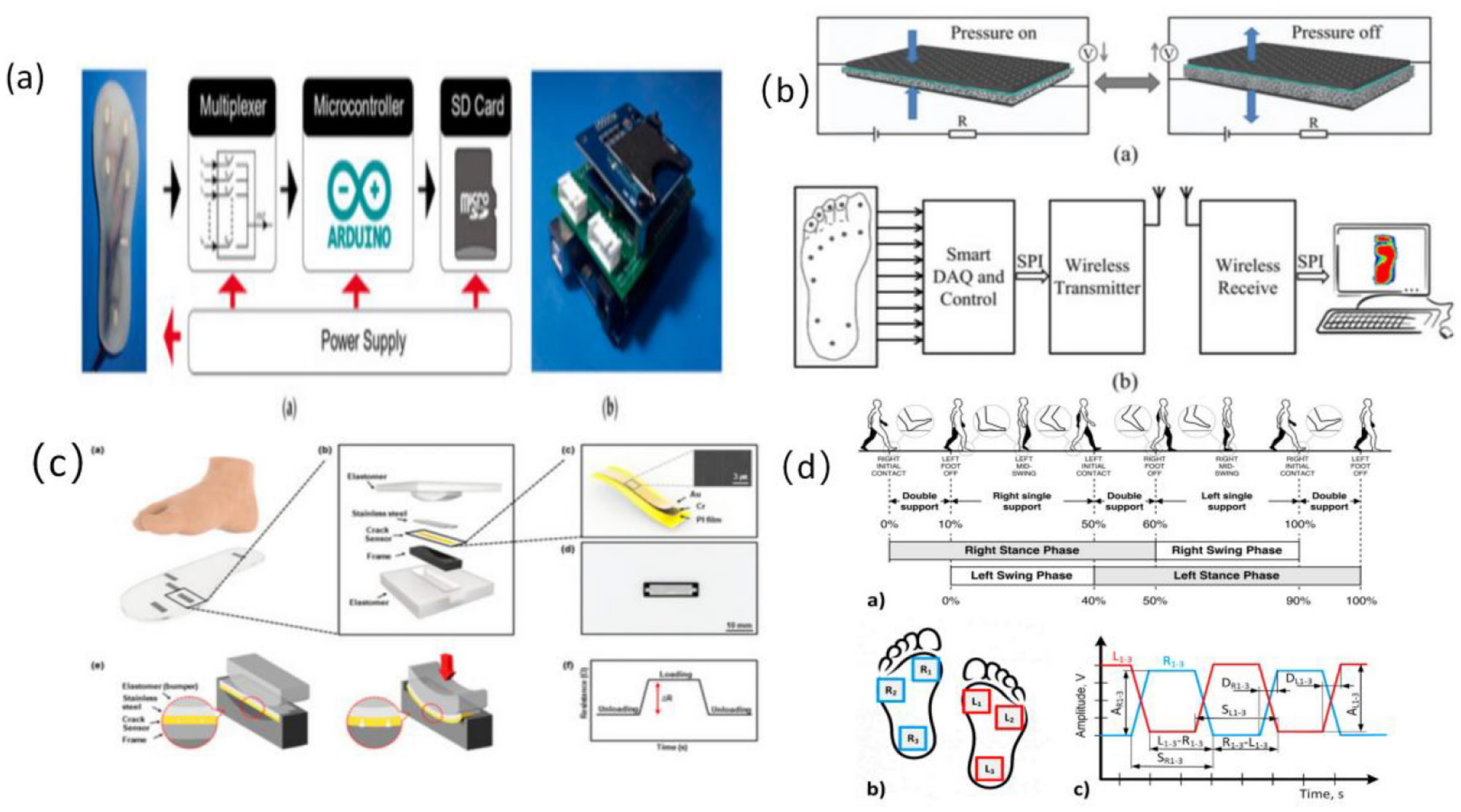
Structure of foot pressure sensor and signal feature extraction workflow. **(a)** Diagram of the electrical system of the plantar pressure receptor (106), copyright (2021), with permission from MDPI. **(b)** Proposed plantar pressure dynamic measurement system (107), copyright (2017), with permission from MDPI. **(c)** Schematic and principle of foot plantar pressure (45), copyright (2019), with permission from MDPI. **(d)** Scheme of human gait analysis (40), copyright (2021), with permission from MDPI.

Plantar pressure sensors reconstruct the center of pressure (CoP) trajectory and support patterns throughout the gait cycle by capturing vertical load variations across multiple key regions of the foot. These sensors are widely employed in gait stability assessment and fall-prevention systems for older adults (39). Common device types include flexible piezoresistive elements (e.g., conductive foam, carbon nanocomposites) and capacitive structures (e.g., PDMS dielectric with ITO electrodes), typically embedded within insoles or smart footwear platforms using 8–16 channel arrays to cover high-load zones such as the heel, metatarsal heads, first toe joint, and lateral foot edge (22, 39, 40) (refer to Figure 3).

From an engineering perspective, high-resolution arrays enable cycle-by-cycle pressure mapping (P–T mapping), gait phase identification (stance–swing segmentation), and extraction of symmetry indices (39). Plantar signals demonstrate high sensitivity to subtle gait perturbations, asymmetric support, and reduced propulsion, making them particularly suitable for detecting gait instability caused by age-related degeneration or fear of falling (28, 39). Typical systems operate at a sampling frequency of 50–100 Hz, with channel linearity exceeding 0.95 and drift stability within 5%, ensuring reliable reconstruction of true gait dynamics.

Among the 23 studies reviewed, 5 explicitly evaluated plantar pressure sensors in gait intervention settings. Systems combining ≥8-channel plantar arrays with triaxial IMUs were used to derive cadence, ground contact time, step length variability CV and fear of falling scores (FES-I). After intervention periods averaging 6.5 weeks, CV decreased from 9.6% to 4.1%, step length stability improved by 16.3%−23.1%, and FES-I scores reduced by 13.5%−17.8%, indicating both biomechanical and psychological benefits (22, 28, 31).

In terms of signal processing, plantar systems are often integrated with IMU data to construct support-phase rhythm maps within time windows. Analytical methods include cross-correlation, symmetry quantification, and CoP trajectory modeling using polynomial fitting (39). Several studies have further incorporated machine learning-based fall-risk classifiers [e.g., SVM, random forests (RF)] and linked gait irregularities to emotional stress markers, improving the timeliness and accuracy of fall event prediction.

In summary, plantar pressure sensors, as highly sensitive mechanical input devices, play a pivotal role in older adults gait intervention systems. Their integration with IMUs enhances tracking of both horizontal and vertical stability parameters while providing robust data foundations for early fall risk detection and personalized feedback on abnormal gait patterns. Future directions include improvements in sensor flexibility and comfort, on-device algorithm deployment, and enhanced adaptability to home-based monitoring scenarios, which will significantly accelerate their adoption in remote health management applications (30).

#### 3.2.3 Optoelectronic volumetric sensors for monitoring heart rate rhythm

**Figure 4:**
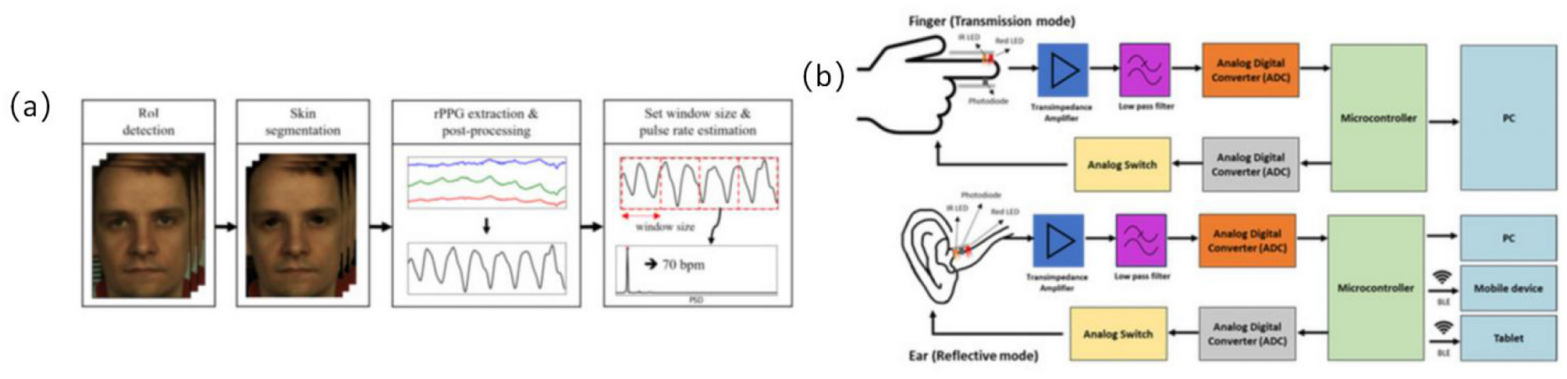
Optoelectronic volumetric sensors signal acquisition path diagram. **(a)** Overview of the proposed remote photoplethysmography (RPPG)-based pulse rate estimation approach (24), copyright (2021), with permission from MDPI. **(b)** Difference of block diagram of in-ear PPG (below) and finger PPG (above) (108), copyright (2023), with permission from MDPI.

Optoelectronic volumetric sensors, commonly known as PPG sensors, continuously and non-invasively monitor heart rate (HR) and HRV by detecting subcutaneous blood volume changes (24, 41, 42). These sensors are widely integrated into wearable devices for recognizing physiological states during exercise interventions in older adults (24, 42). Most PPG sensors are reflective structures embedded within bracelets, wristwatches, or patch devices, enabling real-time cardiovascular response tracking during both rest and dynamic physical activities (41, 43) (refer to Figure 4).

Among the 23 studies included in this review, four utilized wearable devices equipped with PPG modules for ambulatory heart rate monitoring (24, 41–43). For instance, the BioStamp RC wireless patch sensor achieved a Pearson correlation coefficient of up to 0.94 in heart rate detection compared to standard electrocardiogram (ECG), confirming high monitoring accuracy even during indoor treadmill exercise (24, 42). PPG sensors have also demonstrated clinical utility for remote continuous monitoring; another study reported successful capture of heart rate recovery curves during a 6-minute walk test, showing average heart rate recovery times reduced from 87 to 69 s (42, 43). These findings highlight PPG's potential in identifying post-exercise recovery changes.

In summary, as a core physiological sensing module within older adults walking interventions, PPG sensors not only enhance precision in exercise load regulation but also provide cardiovascular safety warnings and personalized rhythmic feedback for older adults. The closed-loop regulatory system created in collaboration with accelerometers, respiratory sensors, and other modalities significantly improves the scientific rigor and practical effectiveness of intervention programs. Consequently, PPG has become one of the critical technical components in developing age-appropriate healthy exercise systems.

#### 3.2.4 Electrodermal sensors for detecting stress response during exercise

**Figure 5:**
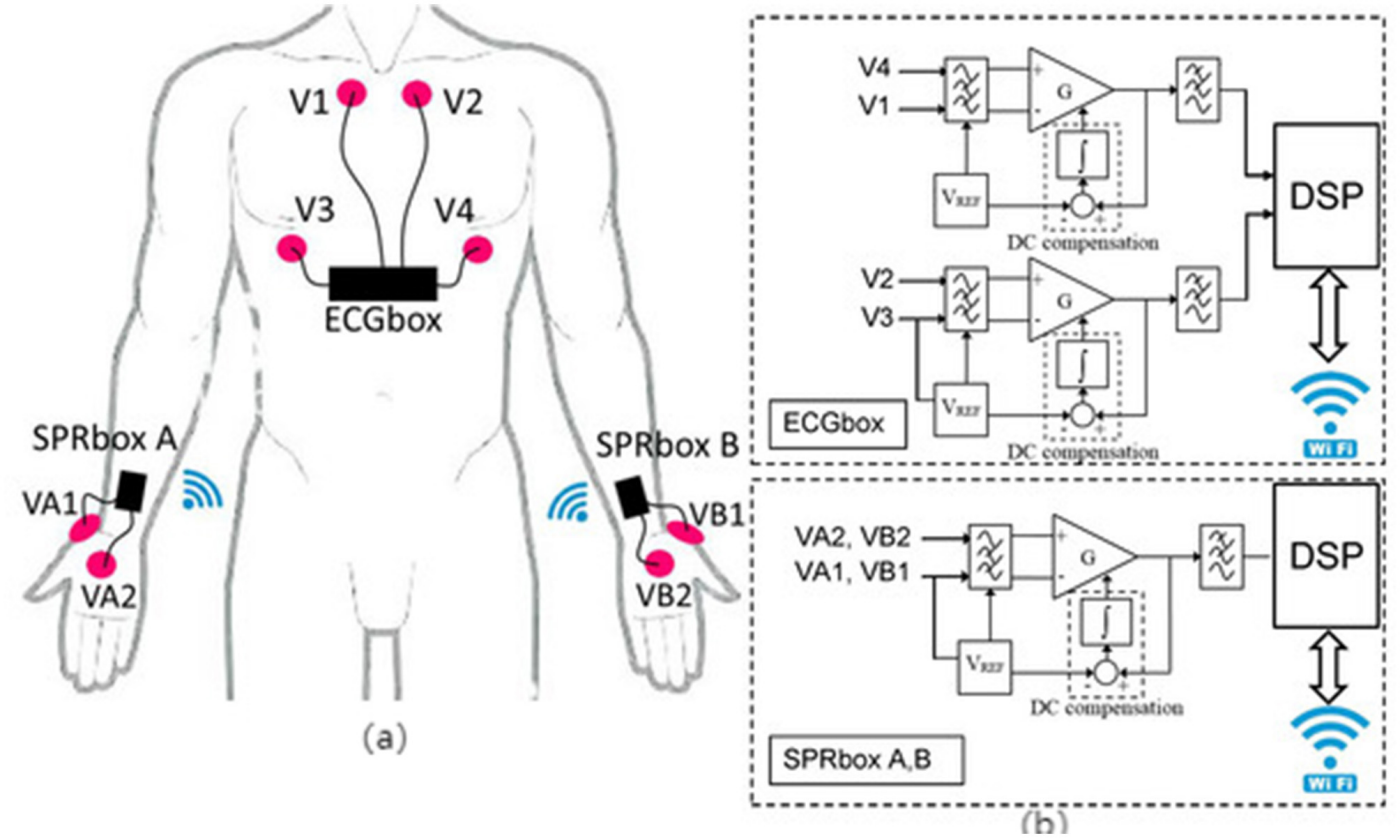
“Schematic of a multi-channel EDA acquisition system and signal conditioning circuit. **(a)** Scheme of sensors and electrodes positioning (63), copyright (2020), with permission from MDPI. **(b)** Electrodermal activity sensor building blocks (63), copyright (2020), with permission from MDPI.

EDA sensors are extensively utilized for monitoring emotional arousal, stress perception, and cognitive load, as they reflect sympathetic nervous system activation through subtle changes in skin conductance (26, 29) (refer to Figure 5). In older adults walking interventions, EDA signals help identify anxiety state changes pre- and post-exercise, cognitive stress responses during dual-task walking, and startle responses during fall scenarios (32). Thus, they effectively capture psychological variables that traditional behavioral metrics cannot fully represent.

Data showed significant elevation of electrodermal activity at the onset of exercise (meanskin conductance response (SCR) frequency increasing from 0.08 to 0.16 beats/s), which subsequently stabilized following a sustained six-week intervention (approximately 37.5% decrease), reflecting adaptive emotional regulation (26, 29, 44). Furthermore, integrating EDA with simultaneous HRV analysis in a bimodal algorithm can detect “hidden stress loads” during non-exercise periods (26, 41, 44), providing critical insights for developing personalized rest and motivation strategies.

In conclusion, electrodermal sensors uniquely complement walking interventions for older adults by effectively detecting emotional activation and psychological stress. Alongside exercise behavior data, EDA can optimize feedback mechanisms, enhance exercise confidence, provide psychological stress warnings, and support personalized rehabilitation and emotionally regulated exercise programs.

### 3.3 Sensor technology supports multidimensional monitoring of intrinsic abilities of the older adults

**Figure 6:**
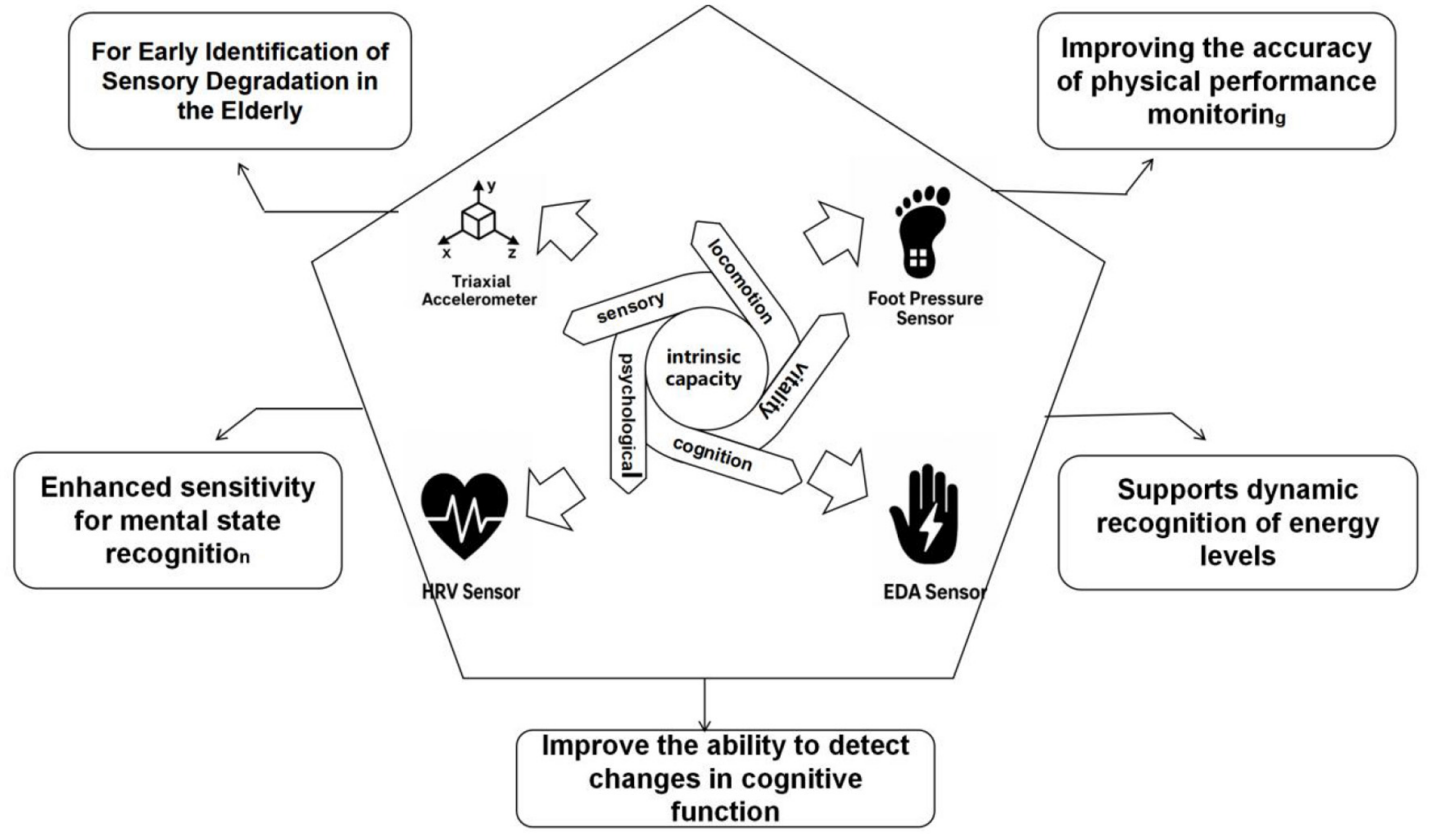
Functional pathways of multi-sensor systems supporting multidimensional intrinsic capacity monitoring in older adults.

Intrinsic capacity reflects core functional dimensions of older adults, including physical, psychological, cognitive, sensory, and vitality domains. Conventional assessment methods lack sensitivity to short-term fluctuations and ecological validity. Wearable sensor technologies enable continuous, high-resolution monitoring of behavioral and physiological signals, offering objective metrics for early risk detection and intervention optimization. The following subsections Figure 6. Multidimensional Sensor Framework for Monitoring Intrinsic Capacity summarize sensor applications across five IC dimensions.

#### 3.3.1 Improves accuracy of physical ability monitoring for the older adults

In Older AdultSensor-based gait monitoring systems can track older adults motor ability changes in natural environments with high frequency and minimal interference, establishing a continuous, quantitative assessment framework for physical capabilities (21, 25, 45). Commonly utilized sensors include plantar pressure sensors and triaxial accelerometers, which respectively capture mechanical support distribution and linear and angular motion characteristics (30, 31). Plantar pressure sensors measure vertical pressure changes on support surfaces using multi-point arrays, reconstructing the COP trajectories and symmetry metrics (22, 28, 30). Triaxial accelerometers identify dynamic movement patterns, such as initiating, moving, and stopping, reflecting daily motor performance levels (22, 32). These integrated systems offer reliable multidimensional data, supporting personalized intervention planning and long-term capability tracking.

Out of 23 studies reviewed, 14 specifically focused on wearable sensor interventions or monitoring with physical capability as a core metric. Eleven studies employed combined plantar pressure sensors and accelerometers for comprehensive monitoring of step length, step frequency, and gait CV (31, 33). Interventions typically lasted 6–12 weeks, with some utilizing dual-task walking experiments across multiple scenarios (22). Results demonstrated notable improvements, including increased average step length (from 0.47 m to 0.56 m), reduced step-frequency CV by 3.8%−6.4%, and significant enhancements in step stability, particularly when feedback mechanisms were employed (25, 36, 39). Additionally, three studies incorporated gait cost indices and power spectral density (PSD) analyses to assess muscular engagement and gait efficiency, finding energy expenditure reductions between 8.5%−13.2% among older adults guided by rhythm-based interventions (35, 36, 46). Moreover, combining plantar feedback with rhythmic interventions enhanced the support symmetry index from 0.76 to 0.89 and improved 6-meter walking speed by ~0.11–0.16 m/s in frail older adults populations (25, 28, 32), confirming that sensor-assisted training reliably boosts lower-limb motor coordination.

In summary, wearable sensor systems significantly enhance monitoring accuracy of older adults physical capability through detailed biomechanical data acquisition. These systems have become essential components of daily behavioral assessments, rehabilitation monitoring, and fall prevention frameworks. Their ability to quantify support symmetry, gait rhythm, and dynamic stability provides the technical foundation for scientifically designing personalized exercise prescriptions and functional training programs.

#### 3.3.2 Enhance sensitivity of mental state recognition in older adults

EDA and HRV sensing modules represent primary technological solutions for recognizing mental stress and emotional arousal during physical activity in older adults (24, 26, 29). EDA sensors capture subtle skin-conductivity changes driven by sweat gland activity (26, 29). HRV monitoring uses PPG or ECG to analyze heart rate interval variations, often employing frequency-domain indices (LF/HF ratio) to assess autonomic nervous system tension (24, 42). These technologies are typically combined into bimodal systems to assess pre-exercise anxiety, in-task stress responses, and post-exercise emotional recovery.

These studies integrated multimodal sensing and machine learning to investigate stress recognition and regulation mechanisms (23, 29). During intervention phases, EDA and HRV signals were employed to train SVM, RF, and deep neural network (DNN) models, effectively classifying high- and low-stress states (23, 26). Intervention outcomes revealed significant improvements after 8 weeks of cognitive training, including a 31.2% reduction inSCR frequency, increased meanRR intervals (from 806 ms to 861 ms), elevated Standard Deviation of Normal to Normal RR Intervals (SDNN) values (from 34.7 ms to 44.1 ms), and over 15.6% improvement in RMSSD. These findings highlight substantial enhancements in autonomic nervous system regulation (23, 29) Multimodal AI-driven models incorporating EDA, HRV, and behavioral data achieved classification accuracies of 88.9% (AUC = 0.92), representing an approximately 18.7% improvement compared to single-channel EDA or HRV models (23, 29).

In summary, dual-channel EDA and HRV sensing technologies sensitively detect stress states and emotional fluctuations in older adults individuals during walking tasks. Their robust algorithm integration and scalability highlight promising applications in personalized intervention feedback and cognitive risk alerts, positioning them as valuable components of future older adults technologies.

#### 3.3.3 Supports dynamic identification of vitality levels of older adults

Vitality serves as a core indicator for assessing the physical and mental energy reserves, exercise recovery capability, and daily activity tolerance among older adults individuals, directly influencing their persistence and willingness to re-engage in exercise interventions. Sensor systems continuously collect behavioral and physiological dual-channel data, establishing dynamic monitoring models with high temporal resolution to detect subtle, non-subjective fatigue accumulation and vitality fluctuations, facilitating early identification of physical decline risks (24, 25, 42).

Devices combining triaxial accelerometers with PPG sensors recorded average daily steps, moderate-to-vigorous physical activity (MVPA) durations, and post-exercise HRR over intervention periods lasting 4–8 weeks (21, 24, 43). These studies demonstrated that objective improvements in vitality could be effectively identified through multi-parameter sensor fusion systems.

Individual exercise tolerance and recovery capacities were notably enhanced. Additionally, three studies compared dynamic energy expenditure (measured in METs or kcal/day using accelerometers) with vitality scores derived from the SF-36 quality-of-life questionnaire, revealing significant correlations (21, 34, 47). These findings confirm the practical feasibility of using quantitative sensor indicators as objective substitutes for subjective vitality assessments (21, 25, 38). In a study involving healthy older women, accelerometer-measured MVPA segments of ≥10 min significantly predicted performance in 5 sit-to-stand tests and 6-min walk distances, highlighting the synergistic relationship between enhanced exercise capacity and improved vitality (34).

In summary, integrated sensor systems combining HRR, SFV, MVPA, and EE parameters provide excellent temporal sensitivity and data interpretability, comprehensively capturing dynamic vitality fluctuations in older adults under natural conditions. Such systems significantly contribute to personalized load management, exercise rhythm optimization, and motivation for continued exercise participation, laying a robust foundation for developing precise and age-friendly vitality monitoring and intervention programs.

#### 3.3.4 For early identification of sensory degradation in the older adults

The degradation of sensory functions, especially vision and vestibular sensation, is a key factor limiting the safety of gait and path planning ability of the older adults. In particular vestibular impairment compromises dynamic balance and postural stability, critical for functional autonomy in older adults (32).In walking interventions, sensory impairments often lead to slow steering, poor spatial orientation and delayed environmental response, significantly increasing the risk of falls (28, 32, 45). Sensor technology provides an objective pathway to identify potential signals of sensory degradation through continuous monitoring of gait rhythm, acceleration fluctuations and head posture changes, and can assist in the individual adaptation of intervention programmes.

A total of five of the included review studies used an integrated system of IMUs (3-axis acceleration + gyroscope) with multipoint plantar pressure sensors to monitor gait performance in sensory decline groups (21, 30, 31). Three of them focused on walking behavior under simulated low light or complex path conditions, and showed that the CV of gait increased by about 38.4% and the step length symmetry index decreased by more than 18% in sensory impaired individuals, suggesting that their spatial sense is reduced and their path control is weakened when they have insufficient visual input (28). Two other analyses combining plantar center of pressure trajectory (COP) and acceleration fluctuation metrics to analyze steering co-ordination and path deviation rates found that older adults with vestibular dysfunction had significantly higher values of steering phase deviation than the control group (21, 22, 31). These deviations correlate strongly with vestibular deterioration, reflecting impaired sensorimotor integration and postural compensation strategies, which are essential for safe turning and adaptive locomotion.

In summary, the 3-axis acceleration and plantar pressure synergistic sensing system has the engineering feasibility to identify the risk of sensory function degradation in older adults, and in particular, it demonstrates higher sensitivity in identifying micro-signals such as steering slowness, support deviation and path instability. This multimodal approach enables real-time monitoring of sensory-related gait disturbances in ecologically valid settings, and provides critical inputs for early screening, personalized fall-prevention strategies, and adaptive rehabilitation protocols. It is of great practical value for early screening, personalized intervention design and fall risk prediction.

#### 3.3.5 Improves detection of cognitive function changes in the older adults

Cognitive functioning directly impacts path planning, rhythmic control, and multitasking during brisk walking in older adults Multimodal sensor systems offer dynamic and objective methods for early detection of MCI, capturing synchronized behavioral rhythms and physiological fluctuations (23, 36). These systems effectively monitor rapid and subtle changes often missed by traditional questionnaires by integrating movement-related and physiological signals, enabling longitudinal tracking of cognitive dynamics under ecologically valid and task-relevant conditions.

Currently, multimodal combinations of triaxial accelerometers, HRV, and EDA sensors are widely employed for cognitive assessments (24, 26, 29). Accelerometers extract gait variability, rhythmic control, and responsiveness to speed changes; HRV measures autonomic nervous system regulation; and EDA indicates stress levels and emotional arousal (21, 26, 29). Together, these signals reflect the integration of motor, autonomic, and affective pathways involved in cognitive control. In dual-task gait scenarios, these systems simultaneously collect behavioral and physiological signals, enabling analysis of executive dysfunction and neural state alterations associated with cognitive loads in individuals with MCI (23, 32).This is particularly valuable in detecting early-stage impairments, where overt clinical symptoms may be absent but compensatory mechanisms such as increased gait variability—begin to emerge.

Numerous reviewed studies utilized these sensor combinations to assess cognitive load and evaluate intervention effects in older adults (23, 29). For example, during dual-task walking, individuals with MCI exhibited increased gait variability (higher CV values) and significantly reduced HRV parameters, such as SDNN, reflecting heightened cognitive interference on motor control systems (23, 32). Additionally, comparisons of electrodermal activity pre- and post-cognitive training indicated reduced SCR frequency and increased root mean square of successive differences (RMSSD) values, suggesting decreased stress levels and improved autonomic nervous function (23, 26, 29). These physiological shifts provide objective, quantifiable evidence of cognitive improvement, and may help tailor interventions based on real-time feedback rather than relying solely on subjective reporting.

In summary, multimodal sensor platforms demonstrate high sensitivity and engineering adaptability for identifying MCI risks and assessing cognitive regulatory capacity in older adults. Compared to static questionnaire-based assessments, sensor-based approaches provide real-time, high-frequency monitoring in dynamic contexts, offering valuable insights for optimizing cognitive interventions and developing effective risk-warning mechanisms. They also promote the development of closed-loop systems for proactive, personalized cognitive care in aging populations.

## 4 Discussions

### 4.1 Multimodal sensor systems enhance detection and monitoring accuracy and effectiveness

**Figure 7:**
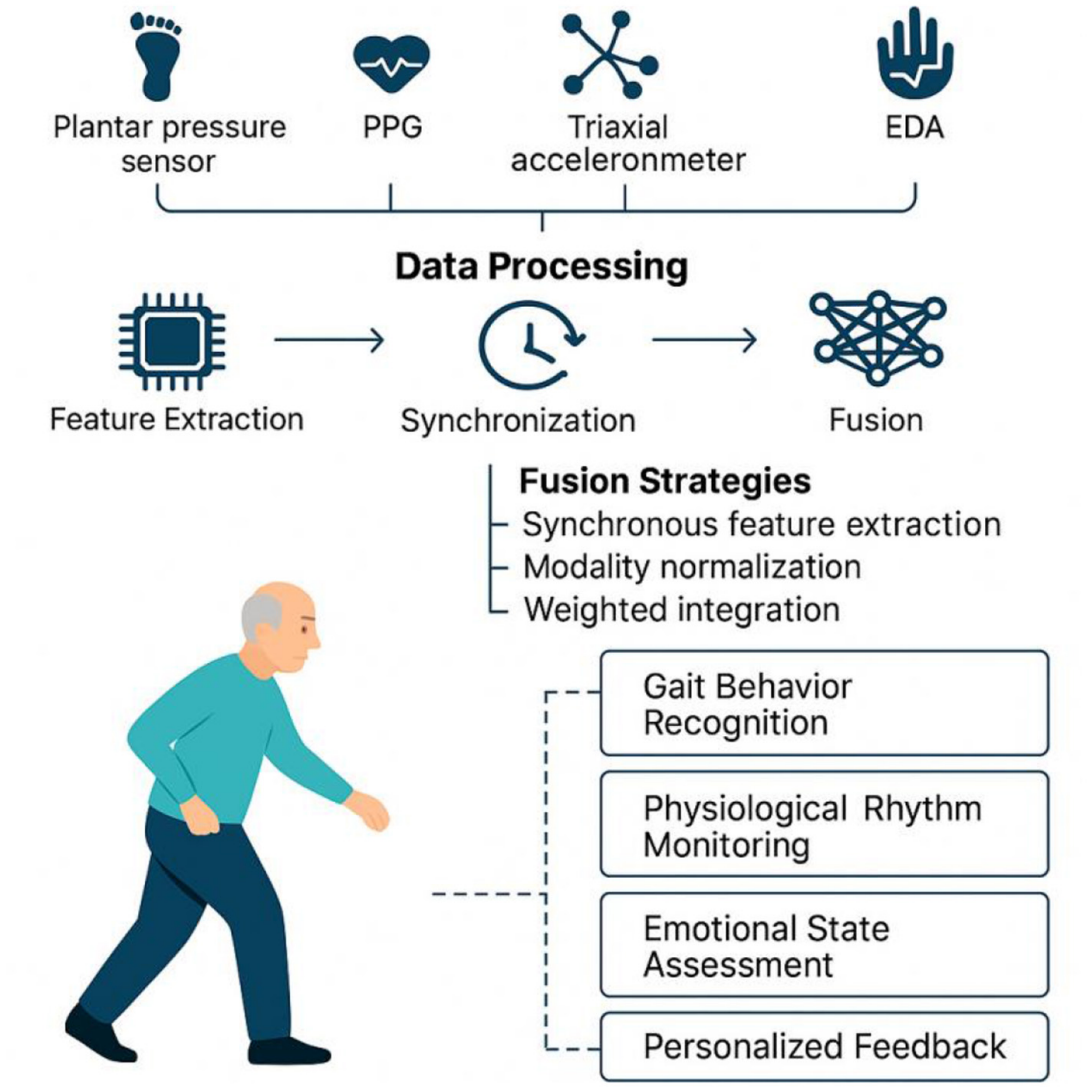
Multimodal sensor fusion framework for behavioral and physiological state monitoring in older adults.

A systematic review of 23 empirical studies demonstrates that employing multimodal sensing systems in walking interventions for older adults significantly enhances monitoring coverage and feedback accuracy across behavioral, physiological, and psychological dimensions—particularly in complex scenarios such as gait recognition, physiological rhythm monitoring, and mood fluctuation assessment (1, 48, 49) (refer to Figure 7). Compared to single-sensor solutions, multimodal systems effectively bridge the informational gaps between motor behavior, cognitive function, and emotional state by integrating multiple sensory channels. This integration enables real-time detection of older adults' dynamic conditions and facilitates personalized, interactive intervention adjustments (29, 50, 51). As a result, these systems substantially improve the responsiveness and individualization of intervention programs, sustain user engagement, and strengthen the long-term efficacy of interventions.

#### 4.1.1 Highly integrated architecture enhances system stability and response efficiency

Multimodal sensing systems usually adopt highly modular and integrated hardware architecture, and through the collaborative deployment of multiple sensors, they can achieve comprehensive capture of the walking behavior, physiological changes and environmental interactions of the older adults. Typical configurations include plantar pressure sensors and IMUs for stability and gait rhythm analysis, PPG sensors combined with triaxial accelerometers for monitoring heart rate load and activity intensity, and EDA and HRV sensors for detecting emotional stress and fearful states (21, 30, 52, 53). The collaborative data acquisition between these hardware modules is achieved through a unified clock synchronization mechanism, and relies on low-power wireless protocols such as BLE 5.0 to ensure stable communication and data integrity of multiple sensing sources.

At the system response level, the introduction of edge computing unit has become a key means to improve the real-time and intelligent level of the system. By realizing feature extraction, preliminary anomaly screening and feedback generation at local nodes, the system can effectively reduce data latency, alleviate the network transmission burden, and improve the feedback efficiency in emergency situations (54–56). For example, for the detected sudden change in gait, sudden increase in heart rate, or violent fluctuations in mood, the edge node can instantly generate alert signals to warn the user through vibration, voice, or light effects.

This type of highly integrated, low-latency design not only improves data processing efficiency and system stability, but also contributes to the long-term operation of the device in real-world scenarios and the maintenance of user stickiness. Studies have shown that this type of system has significant advantages in improving exercise duration, rhythm stability and subjective sense of security, which is an important technical guarantee to promote the standardization of daily exercise behavior and maximize the effectiveness of intervention for the older adults (57–59).

#### 4.1.2 Adaptive algorithms and multimodal data fusion to enhance individual recognition accuracy

In multimodal sensing systems, the scientific rigor and adaptability of the data fusion strategy directly influence the recognition performance and feedback accuracy for complex behavioral states in older adults. Currently, mainstream approaches include time-window-based synchronous feature extraction, modality normalization, and weighted integration, often implemented through deep learning models such as support vector machines (SVMs), CNNs, and long short-term memory (LSTM) networks. These algorithms are effective in capturing temporal correlations within behavioral dynamics, allowing for joint modeling of gait patterns, rhythmic variations, and emotional fluctuations (60, 61).

To account for inter-individual differences in physiological states and behavioral responses, some studies have introduced adaptive fusion mechanisms (62). These systems dynamically adjust the weight distribution of each sensing channel based on individual-specific parameters, such as resting heart rate, baseline gait rhythm, or galvanic skin response. For instance, in users with unstable gait, the algorithm prioritizes IMU and plantar pressure signals, whereas for emotionally sensitive or anxiety-prone individuals, it increases the processing sensitivity of EDA and HRV inputs (21, 45, 63). This approach substantially enhances the system's ability to adapt to individual characteristics and generalize across diverse populations—making intervention strategies not only “tailored to the individual”but also“evolving with the individual”.

In summary, multimodal sensing systems have demonstrated superior monitoring coverage and accuracy in gait interventions for older adults, enabling comprehensive capture of gait characteristics and behavioral changes. These systems provide a robust technological foundation for personalized and dynamically responsive health interventions. By integrating data from multiple sensing modalities, they offer improved risk assessment, more accurate anomaly detection, and enhanced relevance and timeliness of intervention strategies. Future development should prioritize the co-optimization of hardware and software design, along with the advancement of intelligent and user-friendly feedback mechanisms, to facilitate the transition of such systems from experimental validation to real-world application. Moreover, attention should be given to wearability, energy efficiency, and behavioral adaptability to deliver an intelligent health support solution that is continuous, convenient, and focused on behavioral guidance for older adults.

### 4.2 Prospects: focus on accurate identification, personalized modeling, and intelligent feedback

With ongoing advancements in sensor hardware, low-power communication protocols, and artificial intelligence algorithms, older adults walking intervention systems are evolving from single-indicator detection to comprehensive, multidimensional state assessment (64). Future research should focus on three key dimensions: multimodal perception fusion, personalized intervention modeling, and interactive feedback mechanism optimization, to enhance the system's accuracy, adaptability, and user experience (65, 66).

#### 4.2.1 Accurate identification

Multimodal perception fusion is expected to significantly enhance the system's recognition accuracy and contextual awareness of complex behavioral states in older adults. Existing studies have shown that multiple sensing device such as plantar pressure sensors, triaxial accelerometers, PPG, and EDA can be synchronized to capture various state features, including gait rhythm, heart rate variability, and emotional stress responses (67–69). Future research should further address technical challenges in multi-sensor data fusion, including asynchronous data acquisition, transmission delays, and noise interference (70). Additionally, researchers should explore real-time multi-source data co-processing architectures, develop advanced algorithms for feature-level and decision-level fusion, and optimize time synchronization mechanisms to overcome the limitations of single-sensor systems, such as missing data, false alarms, and pose dependency (71, 72). These advancements will enable more accurate and real-time monitoring and early warnings for common risks among older adults, including fall detection, fatigue or overload identification, and gait abnormalities.

#### 4.2.2 Personalized modeling

Personalized intervention modeling will be central to enhancing the relevance and effectiveness of intervention systems. The older adults population is highly heterogeneous in terms of physiological status, cognitive function, perceptual ability, and motivation for exercise, leading to widely varied responses to the same intervention programs (73). Traditional standardized intervention models often fail to adequately address this variability, resulting in unsustainable outcomes. Therefore, future research should enhance dynamic monitoring and build individualized models based on personal parameters such as resting heart rate, gait symmetry, skin conductance frequency, and daily activity capabilities (74). By leveraging advanced machine learning and deep learning, adaptive intervention models can be developed to deliver precise, personalized feedback and strategy adjustments tailored to specific contexts and timeframes (75).ultimately supporting sustained participation in walking and enhancing older adults' confidence and ability in managing their health.

#### 4.2.3 Intelligent feedback

Developing intelligent and context-aware feedback mechanisms will be crucial for improving user engagement and adherence. Current systems often rely on static charts or text notifications, offering limited interactivity and delayed feedback, which fail to meet the older adults' need for immediate interaction and sustained motivation (76, 77). Future research should explore richer multimodal interactions—such as personalized voice prompts, adaptive vibrations, visual animations, and haptic feedback—and incorporate context-aware elements to deliver timely, user-specific feedback (78, 79). In addition, a closed-loop system of real-time data sharing and feedback involving family members, caregivers, and healthcare professionals should be developed to foster a collaborative health management ecosystem (80). This would promote data transparency, enable personalized interventions, and support proactive health risk management and precise behavioral intervention.

In summary, future walking intervention systems for older adults should center on three core technologies: accurate identification, personalized modeling, and intelligent feedback. They should closely align with user needs and promote the evolution of motion sensors from mere data collectors to intelligent health management tools. This can not only slow functional decline and reduce fall risks among older adults, but also offer essential technical and theoretical support for implementing active healthy aging strategies.

### 4.3 Limitations: design rigor, inclusiveness, validation, deployment, ethics in older adults walking systems

This study systematically integrates empirical research on motion sensor interventions targeting walking behavior among older adults and establishes a logical technical framework encompassing sensor types, functional mechanisms, and intrinsic ability support. However, current evidence still presents critical limitations across five dimensions: design rigor, population inclusiveness, long-term validation, realistic deployment and ethical governance.

#### 4.3.1 Design rigor

Notable heterogeneity exists among the included studies regarding sensor types and functional indicators, limiting direct comparability of research outcomes. Significant variability was observed in sensor devices, sensing parameters (such as sampling frequency and number of measurement channels), and functional modules (e.g., IMU integration, real-time feedback) (81, 82). Some studies relied solely on simple accelerometers or wristband devices, inadequately capturing gait stability, physiological rhythms, and psychological states, thereby limiting the generalizability of intervention effects (74, 83). Future studies should incorporate subclass clustering strategies—such as classification based on device integration or perceptual dimensions—to enhance methodological structure and precision.

#### 4.3.2 Inclusiveness

The study samples exhibit significant selection bias, challenging the representativeness of all older adults. Current research predominantly focuses on urban, cognitively healthy, and technologically adept older populations, neglecting special-needs groups, including mobility-impaired individuals, rural residents, and those averse to technology. Such bias may overestimate technology acceptance and adherence, thereby limiting the practical applicability and policy implications of the findings (84). Expanding participant scope by incorporating diverse demographic factors (e.g., age, gender, geographic location, health status, education, and digital literacy) and conducting detailed subgroup analyses will enhance generalizability and policy relevance (85, 86). Furthermore, user adaptation periods and technical barriers encountered during actual deployment must be considered to improve practical effectiveness.

#### 4.3.3 Validation

Existing intervention studies typically have short data collection periods, mostly ranging from 4 to 8 weeks, with few extending to or beyond 12 weeks. Short-term designs may primarily reflect transient behavioral motivation rather than sustained functional improvement or capability reconstruction (87). Moreover, inadequate tracking during the post-intervention or ”off-device” phase restricts reliable assessments of behavioral internalization and long-term self-driven effects of technological interventions. Future interventions should therefore extend the duration of data collection and strengthen follow-up phases to fully elucidate the long-term behavioral change mechanisms (88). Additionally, theoretical explorations into behavioral internalization mechanisms are necessary to clarify how technology facilitates enduring self-driven behaviors among older adults (89).

#### 4.3.4 Deployment

Current research is largely confined to controlled environments, overlooking real-world interference factors such as environmental noise, equipment maintenance, and data transmission stability (90, 91). Specifically, environmental noise may disrupt signal clarity and introduce artifacts in sensor readings; inadequate equipment maintenance can lead to hardware malfunction or calibration drift over time; and unstable data transmission may result in delays, data loss, or incomplete monitoring records (92–94). These factors collectively undermine the accuracy, continuity, and reliability of sensor-based monitoring in real-world aging care settings. In addition, factors such as individual variability in daily activities, the presence of caregivers, and the diversity of living environments can also affect the performance and effectiveness of sensor-based technologies (51, 95).Therefore, future research should emphasize field tests in real-world settings to systematically evaluate technological stability and reliability across diverse practical scenarios (96).Moreover, attention should be given to long-term device use, including wearability, user comfort, and the adaptability of technologies to different living conditions and user needs, to ensure broader applicability and sustained effectiveness of interventions (97, 98).

#### 4.3.5 Ethics

Additionally, the ethical dimensions of deploying sensor-based health monitoring systems, particularly regarding data privacy, user consent, and digital autonomy, remain underexplored in much of the current literature (99). As these systems collect sensitive physiological and behavioral data in real time, they raise critical concerns surrounding data ownership, informed consent clarity, security vulnerabilities, and the potential for surveillance or misuse (100, 101). Future research should not only incorporate transparent ethical frameworks and privacy-by-design protocols, but also actively engage with institutional review boards and stakeholder communities to ensure compliance with regulatory standards and moral obligations (102, 103). Establishing trust, accountability, and user empowerment should be integral to the development and deployment of wearable technologies for older adults.

In conclusion, although existing research initially demonstrates the potential of sensor technology in monitoring and intervening in older adults' walking behavior, continuous improvements in research design, validation of long-term effects, sample representativeness, real-world deployment considerations, and privacy and ethical safeguards are essential. Such enhancements will ensure future sensor-based interventions are more practical, feasible, equitable, universally applicable, and sustainable, thereby effectively contributing to achieving strategic goals for healthy aging.

## 5 Conclusions

This systematic review analyzed 23 empirical studies evaluating motion sensors supporting older adults' walking across population characteristics, device types, and intrinsic capacity dimensions. Results indicated the 60–74 age group had optimal technology acceptance, whereas seniors over 85 required intuitive, user-friendly devices. Plantar pressure sensors effectively assessed gait stability; PPG and HRV sensors enhanced physiological monitoring; accelerometers excelled in behavioral assessment; and EDA sensors sensitively detected emotional stress. Multimodal fusion and AI-driven feedback mechanisms offer significant potential for personalized interventions, tele-rehabilitation, and comprehensive health assessments.

## References

[1] Gianfredi V Nucci D Pennisi F Maggi S Veronese N Soysal P. Aging, longevity, and healthy aging: the public health approach. Aging Clin Exp Res. (2025) 37:125. 10.1007/s40520-025-03021-8 40244306 PMC12006278

[2] Khan HTA Addo KM Findlay H. Public health challenges and responses to the growing ageing populations. Public Health Chall. (2024) 3:e213. 10.1002/puh2.213 40496520 PMC12039680

[3] Koivunen K Schaap LA Hoogendijk EO Schoonmade LJ Huisman M van Schoor N. M. Exploring the conceptual framework and measurement model of intrinsic capacity defined by the World Health Organization: a scoping review. Ageing Res Rev. (2022) 80:101685. 10.1016/j.arr.2022.101685 35830956

[4] Xue Q.-L., Ma L, Chan, P. Intrinsic capacity as a determinant of physical resilience in older adults. J Nutr Health Aging (2021) 25:1006–11. 10.1007/s12603-021-1629-z 34545921 PMC8035602

[5] Lopez-Ortiz S Lista S Penin-Grandes S Pinto-Fraga J Valenzuela PL Nistico R . Santos-Lozano, Defining A, and assessing intrinsic capacity in older people: A systematic review and a proposed scoring system. Ageing Res Rev. (2022) 79:101640. 10.1016/j.arr.2022.101640 35569785

[6] George PP Lun P Ong SP Lim WS. A rapid review of the measurement of intrinsic capacity in older adults. J Nutr Health Aging. (2021) 25:774–82. 10.1007/s12603-021-1622-6 34179933 PMC7966899

[7] Zhou Y Ma L. Intrinsic capacity in older adults: recent advances. Aging Dis. (2022) 13:353–9. 10.14336/AD.2021.0818 35371613 PMC8947834

[8] Jiang X Chen F Yang X Yang M Zhang X Ma X Yan P. Effects of personal and health characteristics on the intrinsic capacity of older adults in the community: a cross-sectional study using the healthy aging framework. BMC Geriatr. (2023) 23:643. 10.1186/s12877-023-04362-7 37817083 PMC10566030

[9] Beard JR Jotheeswaran AT Cesari M Araujo de Carvalho I. The structure and predictive value of intrinsic capacity in a longitudinal study of ageing. BMJ Open. (2019) 9:e026119. 10.1136/bmjopen-2018-026119 31678933 PMC6830681

[10] Richardson DP Foxe JJ Mazurek KA Abraham N Freedman EG. Neural markers of proactive and reactive cognitive control are altered during walking: a Mobile Brain-Body Imaging (MoBI) study. Neuroimage. (2022) 247:118853. 10.1016/j.neuroimage.2021.118853 34954331 PMC8822329

[11] Clark DJ. Automaticity of walking: functional significance, mechanisms, measurement and rehabilitation strategies. Front Hum Neurosci. (2015) 9:246. 10.3389/fnhum.2015.00246 25999838 PMC4419715

[12] Bai X Soh KG Omar Dev RD Talib O Xiao W Cai H. Effect of brisk walking on health-related physical fitness balance and life satisfaction among the elderly: a systematic review. Front Public Health. (2021) 9:829367. 10.3389/fpubh.2021.829367 35174137 PMC8841590

[13] Tully MA Cupples ME Chan WS McGlade K. Young, brisk walking IS, fitness, and cardiovascular risk: a randomized controlled trial in primary care. Prev Med. (2005) 41:622–8. 10.1016/j.ypmed.2004.11.030 15917061

[14] Paraskevoudi N Balci F Vatakis A. “Walking” through the sensory, cognitive, and temporal degradations of healthy aging. Ann N Y Acad Sci. (2018) 1426:72–92. 10.1111/nyas.13734 29741265

[15] Burtan D Joyce K Burn JF Handy TC Ho S Leonards U. The nature effect in motion: visual exposure to environmental scenes impacts cognitive load and human gait kinematics. R Soc Open Sci. (2021) 8:201100. 10.1098/rsos.201100 33614067 PMC7890511

[16] Chen Y Tang H Wang Y Jin C Wang L Miao W Wang X. The effect of complex cognitive context on the dynamic stability during gait initiation in older women. Front Aging Neurosci. (2023) 15:1342570. 10.3389/fnagi.2023.1342570 38274990 PMC10808313

[17] Mao Q Zhang J Yu L Zhao Y Luximon Y Wang H. Effectiveness of sensor-based interventions in improving gait and balance performance in older adults: systematic review and meta-analysis of randomized controlled trials. J Neuroeng Rehabil. (2024) 21:85. 10.1186/s12984-024-01375-0 38807117 PMC11131332

[18] Grimm B Bolink S. Evaluating physical function and activity in the elderly patient using wearable motion sensors. EFORT Open Rev. (2016) 1:112–20. 10.1302/2058-5241.1.160022 28461937 PMC5367538

[19] Kristoffersson A Linden M. A systematic review of wearable sensors for monitoring physical activity. Sensors. (2022) 22:573. 10.3390/s22020573 35062531 PMC8778538

[20] Majumder S. Wearable Systems For Health Monitoring Towards Active Aging. Doctoral dissertation, McMaster University (2020).

[21] Clarke CL Taylor J Crighton LJ Goodbrand JA McMurdo MET Witham MD. Validation of the AX3 triaxial accelerometer in older functionally impaired people. Aging Clin Exp Res. (2017) 29:451–7. 10.1007/s40520-016-0604-8 27435918 PMC5445187

[22] Nakajima K Anzai E Iwakami Y Ino S Yamashita K Ohta Y. Measuring gait pattern in elderly individuals by using a plantar pressure measurement device. Technol Health Care. (2014) 22:805–15. 10.3233/THC-140856 25160000

[23] Delmastro F Di Martino F Dolciotti CJIA. Cognitive training and stress detection in mci frail older people through wearable sensors and machine learning. IEEE Access. (2020) 8:65573–90. 10.1109/ACCESS.2020.2985301

[24] Yu X Laurentius T Bollheimer C Leonhardt S Antink CH. Noncontact monitoring of heart rate and heart rate variability in geriatric patients using photoplethysmography imaging. IEEE J Biomed Health Inform. (2021) 25:1781–92. 10.1109/JBHI.2020.3018394 32816681

[25] Nagayoshi S Oshima Y Ando T Aoyama T Nakae S Usui C . Validity of estimating physical activity intensity using a triaxial accelerometer in healthy adults and older adults. BMJ Open Sport Exerc Med. (2019) 5:e000592. 10.1136/bmjsem-2019-000592 31749982 PMC6830471

[26] Nath RK Thapliyal H. Smart wristband-based stress detection framework for older adults with cortisol as stress biomarker. IEEE Trans Consum Elect. (2021) 67:30–9. 10.1109/TCE.2021.3057806

[27] Tang TB Yeo LW Lau DJH. Activity awareness can improve continuous stress detection in galvanic skin response. In: Proceedings of the Sensors (2014). Valencia: IEEE (2014). p. 1980–1983.

[28] Mickle KJ Munro BJ Lord SR Menz HB. Steele, Foot pain JR, plantar pressures, and falls in older people: a prospective study. J Am Geriatr Soc. (2010) 58:1936–40. 10.1111/j.1532-5415.2010.03061.x 20831725

[29] Kaklauskas A Abraham A Ubarte I Kliukas R Luksaite V Binkyte-Veliene A . A review of AI cloud and edge sensors, methods, and applications for the recognition of emotional, affective and physiological states. Sensors. (2022) 22:7824. 10.3390/s22207824 36298176 PMC9611164

[30] Lee SH Lee HJ Chang WH Choi BO Lee J Kim J . Kim, Gait performance YH, and foot pressure distribution during wearable robot-assisted gait in elderly adults. J Neuroeng Rehabil. (2017) 14:123. 10.1186/s12984-017-0333-z 29183379 PMC5706419

[31] Zhang G Wong DW Wong IK Chen TL Hong TT Peng Y . Plantar pressure variability and asymmetry in elderly performing 60-minute treadmill brisk-walking: paving the way towards fatigue-induced instability assessment using wearable in-shoe pressure sensors. Sensors. (2021) 21:3217. 10.3390/s21093217 34066398 PMC8124239

[32] Howcroft JD Lemaire ED Kofman J McIlroy WE. Analysis of dual-task elderly gait using wearable plantar-pressure insoles and accelerometer. In: Proceedings of the (2014). 36th Annual International Conference of the IEEE Engineering in Medicine and Biology Society. Chicago, IL: IEEE (2014). p. 5003–5006. 10.1109/EMBC.2014.6944748 25571116

[33] Kocuvan P Hrastic A Kareska A Gams M. Predicting a fall based on gait anomaly detection: a comparative study of wrist-worn three-axis and mobile phone-based accelerometer sensors. Sensors. (2023) 23:8294. 10.3390/s23198294 37837123 PMC10575458

[34] Lu Y Li Q Wang W Du L He Q Chen S . Associations between accelerometer-measured physical activity and sedentary behaviour with physical function among older women: a cross-sectional study. BMC Public Health. (2024) 24:1754. 10.1186/s12889-024-19270-7 38956531 PMC11218370

[35] Purwar A Jeong DU Chung WY. Activity monitoring from real-time triaxial accelerometer data using sensor network. In: Proceedings of the 2007 International Conference on Control, Automation and Systems (2007). p. 2402–2406.

[36] Bollaert RE. Motl, Physical RW, Cognitive functions. Physical activity, and sedentary behavior in older adults with multiple sclerosis. J Geriatr Phys Ther. (2019) 42:304–12. 10.1519/JPT.0000000000000163 29200085

[37] Kakita S Watanabe T Yamagishi J Tanaka C Watanabe D Okura . Exploring physical activity levels in patients with cardiovascular disease—a preliminary study. In: Proceedings of the Healthcare (2024). p. 784. 38610206 10.3390/healthcare12070784 PMC11011912

[38] Pradeep Kumar D Toosizadeh N Mohler J Ehsani H Mannier C Laksari K. Sensor-based characterization of daily walking: a new paradigm in pre-frailty/frailty assessment. BMC Geriatr. (2020) 20:164. 10.1186/s12877-020-01572-1 32375700 PMC7203790

[39] Song Z Ou J Shu L Hu G Wu S Xu X Chen Z. Fall risk assessment for the elderly based on weak foot features of wearable plantar pressure. IEEE Trans Neural Syst Rehabil Eng. (2022) 30:1060–70. 10.1109/TNSRE.2022.3167473 35420987

[40] Bucinskas V Dzedzickis A Rozene J Subaciute-Zemaitiene J Satkauskas I Uvarovas V . Wearable feet pressure sensor for human gait and falling diagnosis. Sensors. (2021) 21:5240. 10.3390/s21155240 34372477 PMC8347941

[41] Asher EE Gazit E Montazeri N Mejia-Mejia E Godfrey R Bennett DA . Combining 24-hour continuous monitoring of time-locked heart rate, physical activity and gait in older adults: preliminary findings. Sensors. (2025) 25:1945. 10.3390/s25061945 40293112 PMC11946096

[42] Soares-Miranda L Sattelmair J Chaves P Duncan GE Siscovick DS Stein PK. Mozaffarian, Physical activity D, and heart rate variability in older adults: the Cardiovascular Health Study. Circulation. (2014) 129:2100–10. 10.1161/CIRCULATIONAHA.113.005361 24799513 PMC4038662

[43] Mori K Moriyama S Maruyama H. Visualization of exercise intensity during walking using walk-training robot having photoplethysmographic sensor. In: Proceedings of the (2024). IEEE International Conference on Cyborg and Bionic Systems (CBS). Nagoya: IEEE (2024). p. 17–20.

[44] Choi J Lee S Kim S Kim D Kim H. Depressed mood prediction of elderly people with a wearable band. Sensors. (2022) 22:4174. 10.3390/s22114174 35684797 PMC9185362

[45] Park J Kim M Hong I Kim T Lee E Kim EA Ryu JK . Foot plantar pressure measurement system using highly sensitive crack-based sensor. Sensors. (2019) 19:5504. 10.3390/s19245504 31847062 PMC6960515

[46] Master H Thoma LM Christiansen MB Polakowski E Schmitt LA White DK. Minimum performance on clinical tests of physical function to predict walking 6,000 steps/day in knee osteoarthritis: an observational study. Arthritis Care Res. (2018) 70:1005–11. 10.1002/acr.23448 29045051 PMC5904009

[47] Echevarria-Polo M Marin PJ Pueyo E Ramos Maqueda J Garatachea Variability N . Variability and reliability of the axivity ax6 accelerometer in technical and human motion conditions. Sensors. (2025) 25:2480. 10.3390/s25082480 40285170 PMC12030871

[48] Xefteris V.-R., Tsanousa A, Meditskos G, Vrochidis S, Kompatsiaris I. Performance challenges, and limitations in multimodal fall detection systems: a review. IEEE Sensors J. (2021) 21:18398–409. 10.1109/JSEN.2021.3090454

[49] Ni J Tang H Haque ST Yan Y Ngu AH. A survey on multimodal wearable sensor-based human action recognition. arXiv [preprint] arXiv:2404.15349 (2024). 10.48550/arXiv.2404.15349

[50] Wang Z Yang Z Dong T. A review of wearable technologies for elderly care that can accurately track indoor position, recognize physical activities and monitor vital signs in real time. Sensors. (2017) 17:341. 10.3390/s17020341 28208620 PMC5336038

[51] Jacob Rodrigues M Postolache O Cercas F. Physiological and behavior monitoring systems for smart healthcare environments: a review. Sensors. (2020) 20:2186. 10.3390/s20082186 32290639 PMC7218909

[52] Lachant DJ Light A Offen M Adams J White RJ. Heart rate monitoring improves clinical assessment during 6-min walk. Pulm Circ. (2020) 10:2045894020972572. 10.1177/2045894020972572 33354315 PMC7734514

[53] Dubey TP. AI-driven stress monitoring for older adults: a wearable IOT solution. J Artif Intellig Autonom Intellig Res. (2025) 2:16.

[54] Sundararaman B Buy U Kshemkalyani AD. Clock synchronization for wireless sensor networks: a survey. Ad Hoc Netw. (2005) 3:281–323. 10.1016/j.adhoc.2005.01.002

[55] Bideaux A Zimmermann B Hey S Stork W. Synchronization in wireless biomedical-sensor networks with Bluetooth Low Energy. Curr Direct Biomed Eng. (2015) 1:73–6. 10.1515/cdbme-2015-0019

[56] Ju F Wang Y Yin B Zhao M Zhang Y Gong Y . Microfluidic wearable devices for sports applications. Micromachines. (2023) 14:1792. 10.3390/mi14091792 37763955 PMC10535163

[57] Zhou X Wen S Sun G. Monitoring and analysis of physical exercise effects based on multisensor information fusion. J Sensors. (2022) 2022:1–12. 10.1155/2022/419998535600843

[58] Morone G Papaioannou F Alberti A Ciancarelli I Bonanno M Calabro RS. Efficacy of sensor-based training using exergaming or virtual reality in patients with chronic low back pain: a systematic review. Sensors. (2024) 24:6269. 10.3390/s24196269 39409307 PMC11479095

[59] Daniels K Quadflieg K Robijns J De Vry J Van Alphen H Van Beers R . From steps to context: optimizing digital phenotyping for physical activity monitoring in older adults by integrating wearable data and ecological momentary assessment. Sensors. (2025) 25:858. 10.3390/s25030858 39943497 PMC11820068

[60] Yadav SK Tiwari K Pandey HM Akbar SA. A review of multimodal human activity recognition with special emphasis on classification, applications, challenges and future directions. Knowl-Based Syst. (2021) 223:106970. 10.1016/j.knosys.2021.106970

[61] Gao Z Wang Y Chen J Xing J Patel S Liu X Shi Y. MMTSA Proceedings of the ACM on Interactive, Mobile, Wearable and Ubiquitous Technologies. (2023). p. 1–26. 10.1145/3610872 39558922 PMC11571898

[62] Novak D Mihelj M Munih M. A survey of methods for data fusion and system adaptation using autonomic nervous system responses in physiological computing. Interact Comp. (2012) 24:154–72. 10.1016/j.intcom.2012.04.003

[63] Affanni A. Wireless sensors system for stress detection by means of ecg and eda acquisition. Sensors. (2020) 20:2026. 10.3390/s20072026 32260321 PMC7181292

[64] Qiu Y He Y Ying Y Ma X Zhou H Liang K. Multimodal information fusion detection of fall-related disability based on video images and sensing signals. Appl Intellig. (2025) 55:372. 10.1007/s10489-024-06193-4

[65] Al-Saadawi HFT Das B Das R. A systematic review of trimodal affective computing approaches: text, audio, and visual integration in emotion recognition and sentiment analysis. Expert Syst Appl. (2024) 2024:124852. 10.1016/j.eswa.2024.124852

[66] Hussain J Ul Hassan A Muhammad Bilal HS Ali R Afzal M Hussain S . Model-based adaptive user interface based on context and user experience evaluation. J Multimodal User Interf. (2018) 12:1–16. 10.1007/s12193-018-0258-2

[67] Razak AHA Zayegh A Begg RK Wahab Y. Foot plantar pressure measurement system: A review. Sensors. (2012) 12:9884–912. 10.3390/s120709884 23012576 PMC3444133

[68] Temko A. Accurate heart rate monitoring during physical exercises using PPG. IEEE Trans Biomed Eng. (2017) 64:2016–24. 10.1109/TBME.2017.2676243 28278454

[69] Setz C Arnrich B Schumm J La Marca R Tröster G Ehlert U. Discriminating stress from cognitive load using a wearable EDA device. IEEE Trans Inform Technol Biomed. (2009) 14:410–7. 10.1109/TITB.2009.2036164 19906598

[70] Kong L Peng X Chen Y Wang P Xu M. Multi-sensor measurement and data fusion technology for manufacturing process monitoring: a literature review. Int J Extreme Manufact. (2020) 2:022001. 10.1088/2631-7990/ab7ae6

[71] Singh A Rehman SU Yongchareon S Chong PHJ. Sensor technologies for fall detection systems: a review. IEEE Sensors J. (2020) 20:6889–919. 10.1109/JSEN.2020.2976554

[72] Ankalaki S. Simple to complex, single to concurrent sensor based human activity recognition: perception and open challenges. IEEE Access. (2024). 10.1109/ACCESS.2024.3422831

[73] Pichierri G Wolf P Murer K de Bruin ED. Cognitive and cognitive-motor interventions affecting physical functioning: a systematic review. BMC Geriatr. (2011) 11:1–19. 10.1186/1471-2318-11-29 21651800 PMC3147016

[74] Khusainov R Azzi D Achumba IE Bersch SD. Real-time human ambulation, activity, and physiological monitoring: taxonomy of issues, techniques, applications, challenges and limitations. Sensors. (2013) 13:12852–902. 10.3390/s131012852 24072027 PMC3859040

[75] Kothinti RR. Deep learning in healthcare: Transforming disease diagnosis, personalized treatment, and clinical decision-making through AI-driven innovations. World J Adv Res Rev. (2024) 24:2841–56 10.30574/wjarr.2024.24.2.343540216258

[76] Panagopoulos C Menychtas A Tsanakas P. Maglogiannis I. Increasing usability of homecare applications for older adults: a case study. Designs. (2019) 3:23. 10.3390/designs3020023

[77] Johnson J Finn K. Designing User Interfaces for an Aging Population: Towards Universal Design. Burlington, MA: Morgan Kaufmann. (2017).

[78] Dritsas E Trigka M Troussas C. Mylonas, multimodal interaction p, interfaces, and communication: a survey. Multimodal Technol Interact. (2025) 9:6. 10.3390/mti9010006

[79] op den Akker H Jones VM Hermens HJ. Tailoring real-time physical activity coaching systems: a literature survey and model. User Model User-Adapted Interact. (2014) 24:351–92. 10.1007/s11257-014-9146-y

[80] Sharma SV McPherson H Sandoval M Goodman D Paret C Mahata K . Design and framework of a technology-based closed-loop referral project for care coordination of social determinants of health. Popul Health Manag. (2024) 27:390–6. 10.1089/pop.2024.0129 39605191

[81] Prasanth H Caban M Keller U Courtine G Ijspeert A Vallery H . Wearable sensor-based real-time gait detection: a systematic review. Sensors. (2021) 21:2727. 10.3390/s21082727 33924403 PMC8069962

[82] Panebianco GP Bisi MC Stagni R Fantozzi S. Analysis of the performance of 17 algorithms from a systematic review: Influence of sensor position, analysed variable and computational approach in gait timing estimation from IMU measurements. Gait Posture. (2018) 66:76–82. 10.1016/j.gaitpost.2018.08.025 30170137

[83] Bowman T Gervasoni E Arienti C Lazzarini SG Negrini S Crea S . Wearable devices for biofeedback rehabilitation: a systematic review and meta-analysis to design application rules and estimate the effectiveness on balance and gait outcomes in neurological diseases. Sensors. (2021) 21:3444. 10.3390/s21103444 34063355 PMC8156914

[84] Sieverink F Kelders SM van Gemert-Pijnen JE. Clarifying the concept of adherence to eHealth technology: systematic review on when usage becomes adherence. J Med Intern Res. (2017) 19:e402. 10.2196/jmir.8578 29212630 PMC5738543

[85] Mehl KR Morain SR Largent E. A. The importance of including underserved populations in research. Pharmaceut Med. (2025) 2025:1–13. 10.1007/s40290-025-00562-1 40169528 PMC11980435

[86] Munger K Gopal I Nagler J Tucker JA. Accessibility and generalizability: are social media effects moderated by age or digital literacy? Res Politi. (2021) 8:20531680211016968. 10.1177/20531680211016968

[87] Done A Voss C Rytter NG. Best practice interventions: Short-term impact and long-term outcomes. J Operat Managem. (2011) 29:500–13. 10.1016/j.jom.2010.11.007

[88] Hill KG Woodward D Woelfel T Hawkins JD Green S. Planning for long-term follow-up: strategies learned from longitudinal studies. Prevent Sci. (2016) 17:806–18. 10.1007/s11121-015-0610-7 26453453 PMC5337427

[89] Jang S.-b, Kim M. Digital fitness revolution: User perspectives on Fitbit's role in health management. Behav Sci. (2025) 15:231. 10.3390/bs15020231 40001862 PMC11851486

[90] Jesus G Casimiro A Oliveira A. A survey on data quality for dependable monitoring in wireless sensor networks. Sensors. (2017) 17:2010. 10.3390/s17092010 28869505 PMC5620495

[91] Pandey S Chaudhary M Tóth Z. An investigation on real-time insights: enhancing process control with IoT-enabled sensor networks. Discover Intern Things. (2025) 5:29. 10.1007/s43926-025-00124-633652773

[92] De Cheveigné A Simon JZ. Sensor noise suppression. J Neurosci Methods. (2008) 168:195–202. 10.1016/j.jneumeth.2007.09.012 17963844 PMC2253211

[93] Adeyeri MK Mpofu K Kareem B. Development of hardware system using temperature and vibration maintenance models integration concepts for conventional machines monitoring: a case study. J Indust Eng Int. (2016) 12:93–109. 10.1007/s40092-015-0132-8

[94] Wei X Liu Y Gao S Wang X Yue H. An RNN-based delay-guaranteed monitoring framework in underwater wireless sensor networks. IEEE Access. (2019) 7:25959–71. 10.1109/ACCESS.2019.2899916

[95] Stavropoulos TG Papastergiou A Mpaltadoros L Nikolopoulos S Kompatsiaris I. IoT wearable sensors and devices in elderly care: A literature review. Sensors. (2020) 20:2826. 10.3390/s20102826 32429331 PMC7288187

[96] Liu F Panagiotakos D. Real-world data: a brief review of the methods, applications, challenges and opportunities. BMC Med Res Methodol. (2022) 22:287. 10.1186/s12874-022-01768-6 36335315 PMC9636688

[97] Phuyal S Elvas LB Ferreira JC Bista R. Harnessing wearable devices for enhanced long-term care: Opportunities, challenges, future directions. Int J Comp Inform Syst Indust Managem Appl. (2024) 16:13.

[98] Lee J Kim D Ryoo HY Shin BS. Sustainable wearables: wearable technology for enhancing the quality of human life. Sustainability. (2016) 8:466. 10.3390/su8050466

[99] Zhang J Hassandoust F Johnston AC. Privacy in smart health monitoring: a systematic review and research directions. Commun Assoc Inform Syst. (2025) 57:15.40617964

[100] Khatiwada P Yang B Lin JC Blobel B. Patient-generated health data (PGHD): understanding, requirements, challenges, and existing techniques for data security and privacy. J Pers Med. (2024) 14:282. 10.3390/jpm14030282 38541024 PMC10971637

[101] Adeniyi AO Arowoogun JO Okolo CA Chidi R Babawarun O. Ethical considerations in healthcare IT: A review of data privacy and patient consent issues. World J Adv Res Rev. (2024) 21:1660–8. 10.30574/wjarr.2024.21.2.0593

[102] Kisselburgh L Beever J. The ethics of privacy in research and design: Principles, practices, and potential. In: Modern Socio-Technical Perspectives on Privacy. Cham: Springer International Publishing (2022). p. 395–426.

[103] Ikwuanusi UF Adepoju PA Odionu CS. Advancing ethical AI practices to solve data privacy issues in library systems. Int J Multidiscipl Res Updates. (2023) 6:033–44. 10.53430/ijmru.2023.6.1.0063

[104] Duncan S Stewart T Mackay L Neville J Narayanan A Walker C . Wear-time compliance with a dual-accelerometer system for capturing 24-h behavioural profiles in children and adults. Int J Environ Res Public Health. (2018) 15:1296. 10.3390/ijerph15071296 29933548 PMC6069278

[105] Majidzadeh Gorjani O Byrtus R Dohnal J Bilik P Koziorek J Martinek R. Human activity classification using multilayer perceptron. Sensors. (2021) 21:6207. 10.3390/s21186207 34577418 PMC8473251

[106] Ren B Liu J. Design of a plantar pressure insole measuring system based on modular photoelectric pressure sensor unit. Sensors. (2021) 21:3780. 10.3390/s21113780 34072553 PMC8199404

[107] Lou C Wang S Liang T Pang C Huang L Run M . A graphene-based flexible pressure sensor with applications to plantar pressure measurement and gait analysis. Materials. (2017) 10:1068. 10.3390/ma10091068 28891991 PMC5615722

[108] Azudin K Gan KB Jaafar R Ja'afar MH. The principles of hearable photoplethysmography analysis and applications in physiological monitoring-a review. Sensors. (2023) 23:6484. 10.3390/s23146484 37514778 PMC10384007

[109] Lai TF Liao Y Lin CY Huang WC Hsueh MC Chan DC. Moderate-to-vigorous physical activity duration is more important than timing for physical function in older adults. Sci Rep. (2020) 10:21344. 10.1038/s41598-020-78072-0 33288797 PMC7721720

